# MRI findings of nelarabine‐related rhabdomyolysis in a patient with refractory T‐cell acute lymphoblastic leukemia

**DOI:** 10.1002/jha2.705

**Published:** 2023-05-24

**Authors:** Daiki Mukai, Naonori Harada, Ikumi Shibano, Yusuke Kizawa, Hiroshi Shiragami, Atsuko Mugitani, Masayuki Hino

**Affiliations:** ^1^ Department of Hematology Fuchu Hospital Osaka Japan; ^2^ Department of Hematology Graduate School of Medicine Osaka Metropolitan University Osaka Japan

1

A 76‐year‐old man was diagnosed with T‐cell lymphoblastic lymphoma (T‐ALL) and began chemotherapy with hyper‐CVAD/MA (cyclophosphamide, vincristine, doxorubicin, and dexamethasone alternating with methotrexate and cytarabine) [1]. Four courses later, he achieved complete remission, but bone marrow examination after 4 months showed relapsed T‐ALL. Reinduction therapy with L‐asparaginase was initiated, but bone marrow examination again demonstrated recurrent T‐ALL. Thus, he received salvage therapy with nelarabine (1500 mg/m^2^) on days 1 and 3. However, he complained of bilateral thigh pain that was exacerbated by pressure, and that rapidly became incapacitating on day 5. Laboratory findings demonstrated elevated serum creatinine phosphokinase (CK) at 2861 IU/L. Magnetic resonance imaging (MRI) findings showed abnormal bilateral signal intensity of the iliopsoas, rectus femoris, and sartorius. T2‐weighted images and short tau inversion recovery (STIR) images of these muscles depicted diffuse high signal intensity, while T1‐weighted images showed low signal intensity (Figure 1). The patient was diagnosed with nelarabine‐induced rhabdomyolysis since he had no other apparent causes of rhabdomyolysis, including trauma, excessive physical activity, and muscle compression. We discontinued nelarabine and started intravenous hydration. Although his CK level rose to a maximum of 11,299 IU/L on day 7, it gradually normalized without renal dysfunction, and all symptoms resolved on day 9. Thereafter, the patient's relapsed T‐ALL continued to progress and he did not desire further treatment other than best supportive care. On day 20, he succumbed to relapsed T‐ALL despite the control of symptoms associated with nelarabine‐induced rhabdomyolysis.

**FIGURE 1:**
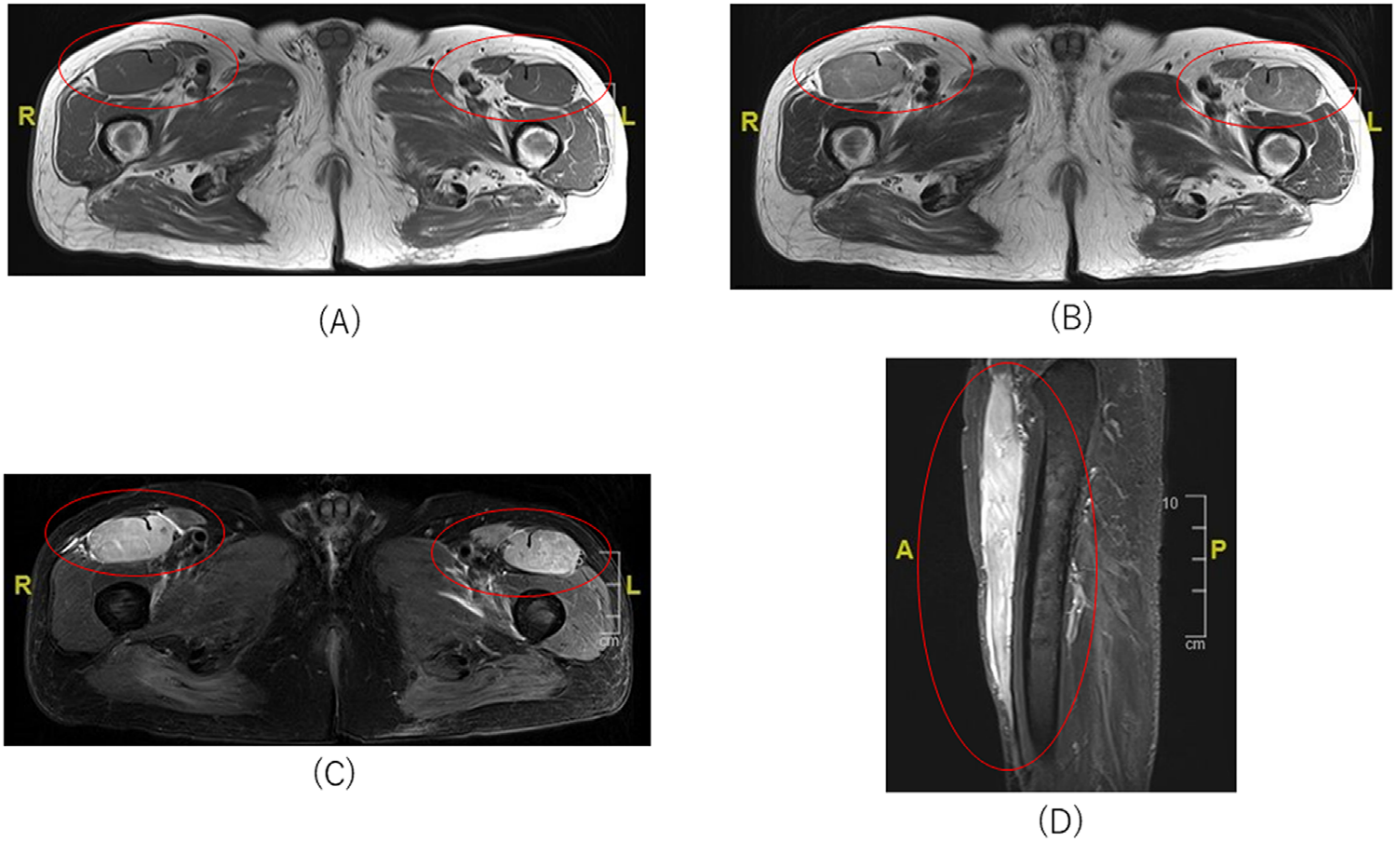
(A) Axial T1‐weighted images show bilateral low signal intensity in the iliopsoas, rectus femoris, and sartorius. (B) Axial T2‐weighted images and (C) axial and (D) sagittal short tau inversion recovery (STIR) images show diffuse, bilateral high signal intensity in the iliopsoas, rectus femoris, and sartorius.

Rhabdomyolysis is a potentially fatal disease with a wide variety of causes, including excessive exercise, trauma, drug abuse, heat stroke, and medicines [2]. It is an infrequent complication caused by treatment with nelarabine [3, 4, 5, 6], a purine analog anticancer drug used in patients with relapsed or refractory T‐ALL [7, 8]. Typical symptoms of rhabdomyolysis include pain, weakness, incapacitation, and swelling of damaged muscles. Nevertheless, it can also present with nonspecific symptoms of fatigue, nausea, and fever‐up, and therefore may be difficult to diagnose during the early phase. Thus, hematologists should comprehensively and objectively evaluate symptoms to differentiate them from hematological malignancy‐related events and other adverse events of chemotherapy. MRI allows clinicians to assess the spread and development of rhabdomyolysis, and its sensitivity for detecting the condition is superior to that of computed tomography and ultrasound. Characteristic MRI findings of damaged muscles in rhabdomyolysis include high signal intensity on T2‐weighted spin echo images and low signal intensity on T1‐weighted images. STIR produces fat‐suppressed images that show a distinctive contrast between normal and rhabdomyolysis‐affected muscles [9].

Our case highlights that MRI is clinically useful for diagnosing rhabdomyolysis, even in patients receiving nelarabine, and for accurately assessing the distribution and extent of muscle lesions.

## AUTHOR CONTRIBUTIONS

Daiki Mukai and Naonori Harada contributed to the concept and design of the manuscript. Ikumi Shibano, Yusuke Kizawa, Hiroshi Shiragami, Atsuko Mugitani, and Masayuki Hino interpreted the results, and reviewed critically and revised the manuscript. All authors read and approved the final version.

## CONFLICT OF INTEREST STATEMENT

The authors declare no conflicts of interest.

## FUNDING INFORMATION

The authors received no specific funding for this work.

## ETHICS STATEMENT

This study was conducted as per the Declaration of Helsinki.

## PATIENT CONSENT STATEMENT

Written informed consent was obtained from the patient for the publication of this study and any identifying images or other personal or clinical details.

## CLINICAL TRIAL REGISTRATION

The authors have confirmed clinical trial registration is not needed for this submission.

## Data Availability

None.
